# The Diagnostic Value of Irisin in Pediatric Patients with Acute Abdominal Pain

**DOI:** 10.1155/2018/3296535

**Published:** 2018-09-24

**Authors:** Fatma Sarac, Sevgi Buyukbese Sarsu, Selman Yeniocak, Kamil Sahin, Esma Yucetas, Dogan Yildirim, Macit Koldas, Ozlem Uzun

**Affiliations:** ^1^Department of Pediatric Surgery, Haseki Research and Education Hospital, Istanbul, Turkey; ^2^Department of Pediatric Surgery, Cengiz Gökcek Obstetrics and Children's Hospital, Gaziantep, Turkey; ^3^Department of Emergency Medicine, Haseki Research and Education Hospital, Istanbul, Turkey; ^4^Department of Pediatrics, Haseki Research and Education Hospital, Istanbul, Turkey; ^5^Department of Biochemistry, Haseki Research and Education Hospital, Istanbul, Turkey; ^6^Department of General Surgery, Haseki Research and Education Hospital, Istanbul, Turkey; ^7^Department of Emergency Medicine, Bagcilar Research and Education Hospital, Istanbul, Turkey

## Abstract

**Objectives:**

Diagnosis of pediatric patients presenting to the Emergency Department with acute abdominal pain is not always easy. The purpose of this study was to investigate the effectiveness of irisin, a peptide hormone with reactivity shown in the appendix and neutrophils, in the differential diagnosis of pediatric patients with acute abdominal pain.

**Methods:**

162 subjects consenting to participate, including 112 patients presenting to the Pediatric Emergency and Pediatric Surgery clinics with acute abdominal pain and 50 controls, were enrolled in the study. Blood was collected from all patients following initial examination for irisin, WBC, and CRP investigation.

**Results:**

Mean irisin levels in cases of acute appendicitis (AA) and perforated appendicitis (PA) were statistically significantly higher compared to nonspecific abdominal pains and the control group. No statistically significant difference was observed in irisin levels between AA and PA cases. WBC and CRP levels were also significantly higher in cases of AA and PA compared to nonspecific abdominal pains.

**Conclusions:**

Differential diagnosis of acute abdominal pains in children and deciding on surgery are a difficult and complex process. Our study shows that irisin can be a useful biomarker in differentiating AA and PA from other acute abdominal pains in children.

## 1. Introduction

Abdominal pain is the most commonly encountered symptom in children in the course of various diseases associated with the intra-abdominal or other systems and on presentation at the Emergency Department. Acute abdominal pain is a term used to describe sudden onset of common clinical symptoms and findings of inflammatory diseases, mostly intra-abdominal but sometimes also in the extra-abdominal region, and requiring surgery [1, 2]. Disease requiring surgery may be present in 5% of patients presenting at the Emergency Department with abdominal pain. Acute appendicitis (AA) is the most common cause of acute abdomen [3–5]. Diagnosis is primarily based on clinical assessment, but the classical symptoms in adults are not generally present in children. Abdominal examination is particularly difficult in very young children since cooperation may not be fully established. Various laboratory parameters, such as WBC, CRP, and neutrophil percentage, and radiological tests such as abdominal ultrasonography (USG) and computed tomography (CT) can assist diagnosis, but none are sufficient for definite diagnosis or to differentiate surgical abdominal pains from other pains. Complete diagnosis is not possible in one in three patients presenting at the Emergency Department with abdominal pain, irrespective of age [3, 4, 6].

Irisin is an exercise hormone derived from skeletal muscle. It is part of the protein fibronectin type III domain containing 5 (Fndc5). Fndc5 enables irisin to be secreted from muscle cells. It is produced in several locations, including fat tissue, the heart, and the salivary glands [7–12]. Studies have shown irisin immunoreactivity in all sites containing adipose tissue. The appendix and neutrophils are among the tissues in which irisin activity has been shown [4, 8].

Studies have been performed with parameters such as CRP, bilirubin, and D-dimer in the differential diagnosis of diseases causing acute abdomen and requiring surgical intervention. This study was planned around the hypothesis that irisin, a peptide hormone and focus of considerable recent interest, may be useful in distinguishing AA from other nonspecific acute abdominal pains.

## 2. Materials and Methods

### 2.1. Patients, Samplings, and Ethical Approval

This research was performed as a single-center, prospective, cross-sectional study. Approval was obtained from the SBU Haseki Training and Research Hospital Ethics Committee (17.2.2016 Number: 233).

The study included a total of 112 patients, aged 2-17 years (mean±SD= 10.0±3.6), who presented at the Pediatric Emergency and Pediatric Surgery clinics due to acute abdominal pain and were definitely diagnosed. A control group was formed of 50 healthy subjects aged 1-17 years (mean±SD=8.0±4.4). Since blood irisin levels can be affected by body mass index (BMI), only cases with BMI values between 20 and 25 were enrolled in the two groups. The parents of all patients were notified of the factors to be evaluated, and informed consent forms were obtained.

Diagnosis was made on the basis of anamnesis, physical examination, biochemical blood tests, and radiological imaging and of surgical specimens from patients undergoing surgery. AA was present in 54 patients, perforated appendicitis (PA) in 16, nonspecific abdominal pain in 34, and other abdominal pains (mesenteric lymphadenitis-MLA (2), acute gastroenteritis-AGE (3), Familial Mediterranean Fever-FMF (1), ovarian cyst (1), and urinary tract infection-UTI (1)) in 8.

### 2.2. Measurements

Human Irisin ELISA kits (Catalog No. CK-E90905, Eastbiopharm, Hangzhou Eastbiopharm Co. Ltd.) were used to determine irisin levels, following the manufacturer's instructions. This kit uses the ELISA technique to detect human irisin with biotin double antibody sandwich technology. First, it adds irisin to the wells which are then coated with monoclonal antibodies and incubated. Anti-irisin antibodies labeled with biotin are then added to combine with streptavidin-HRP and form an immune complex. Unbounded enzymes are removed by washing. The solution turns from blue to yellow under the effect of the acid. Solution shades are correlated with irisin concentrations. Specimen absorbance values were determined on a Biotek ELX800 (Biotek, Winooski, VT, USA) microplate reader at a wavelength of 450 nm. The results were expressed in *μ*g/mL. The minimum detectable level was 0.05 *μ*g/mL. Values were expressed as microgram/mL in calculations converted to nanogram/mL

All WBC levels were measured using a Sysmex XE-2100 hematology analyzer (Sysmex Turkey Diagnostic Systems Ltd., Turkey), and CRP levels were measured using a Beckman Coulter AU680 biochemistry analyzer (Beckman Coulter Inc., Brea, CA, USA).

### 2.3. Statistical Analysis

Statistical analysis was performed using SPSS 15.0 for Windows software. Descriptive data were expressed as number and percentage for categorical variables and mean, standard deviation, minimum, maximum, and median for numerical variables. Comparisons of numerical variables in two independent groups were performed using the Mann–Whitney U test since normal distribution was not established. Comparisons of numerical variables among more than two independent groups were made using the Kruskall Wallis test since normal distribution was not established. Subgroup analyses were performed using the Mann–Whitney U test and were interpreted with Bonferroni correction. Relationships between numerical variables were examined using Spearman correlation analysis since parametric test conditions were not established. Determination of the factor effect was investigated using logistic regression analysis. Alpha significance was set at p<0.05.

## 3. Results

The study included 162 subjects, as 112 patients and 50 healthy control subjects. AA was present in 54 patients (48.2%), nonspecific abdominal pain was in 34 (30.4%), PA was in 16, and other abdominal pains were in 8. The patient group comprised 70 (62.5%) males and 42 (37.5%) females, and the control group comprised 29 (58%) males and 21 (42%) females (Table 1). The mean irisin levels of the groups are shown in Table 2.

The mean irisin values in the patients with AA and PA were statistically significantly higher compared to those in the control group (p=0.001 and p=0.003, respectively). The mean irisin levels of patients with AA and PA were also significantly higher than those in patients with nonspecific abdominal pain (p<0.001 and p=0.002, respectively). There was no significant difference in the mean irisin values between patients with AA and PA (Table 3).

The mean WBC and CRP values of the patient group are shown in Table 4.

The mean WBC and CRP values in patients with AA and PA were significantly higher compared to patients with nonspecific abdominal pain (Table 5).

**Figure 1:**
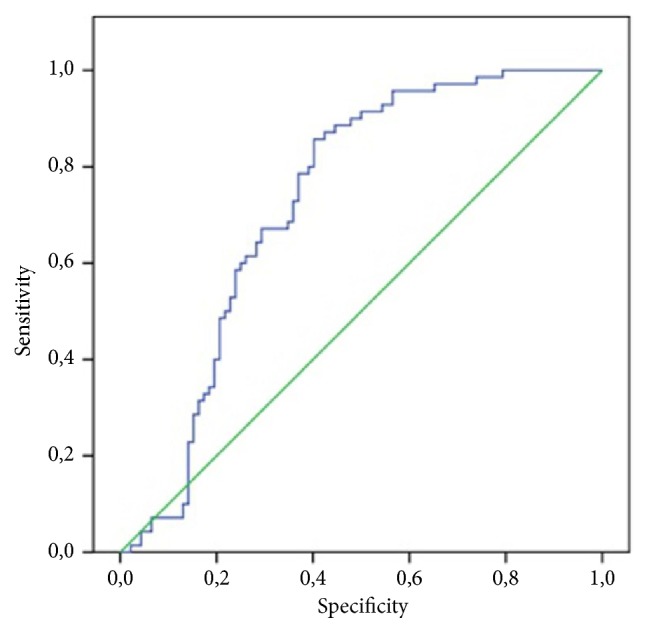
ROC curve for irisin in acute and perforated appendicitis.

Irisin cutoff value ROC analysis was performed for acute and perforated appendicitis (AUC: 0.729 95% CI 0.650-0.807) (Figure 1).

Sensitivity of 80% and specificity of 60.9% were determined at an irisin cutoff value of 8.91 and above for acute-perforated appendicitis in the ROC curve analysis (PPV 60.9%, NPV 80%, accuracy 69.1%).

## 4. Discussion

In our study, the blood irisin levels of patients with AA and PA were higher than those in the nonspecific abdominal pain and control groups. The detection of high blood irisin in the vermiform appendix, a tubular organ, under conditions of infection and perforation suggested that irisin might be used as a marker in the diagnosis of these diseases. From that perspective, Bakal et al.'s study supports our own research. Bakal et al. investigated irisin levels in diseased vermiform appendix tissue examined using both immunohistochemical methods and blood, urine, and saliva irisin levels. They determined significantly higher irisin levels with both methods in patients with AA. They also determined a dramatic increase in blood, urine, and saliva irisin levels after surgery in the patients included in their study. On the basis of their findings, they suggested that irisin can be used, together with an increase in neutrophil levels, as a good marker in AA [4]. While their findings support our own research, the advantage of their study is that Bakal et al. also used immunohistochemical methods to determine irisin levels in appendicitis tissue. The advantage of our study, in contrast, is that we investigated irisin levels in comparison to other abdominal pains. We think that ours being the first study to compare blood irisin levels with other abdominal pains makes it particularly valuable.

Irisin levels have hitherto been principally associated with muscle mass and insulin resistance. Additionally, although irisin's effects have been investigated in cardiovascular diseases, chronic renal failure, some cancers, metabolic diseases, nonalcoholic liver disease, and osteoporosis, irisin has nevertheless been evaluated more as a hormone than as a biochemical marker [9].

Our study shows that irisin can be used as a biochemical marker in AA, a cause of specific abdominal pains. We may attribute the blood irisin level elevation in AA and PA in our study to these organs being tubular organs. Aydın et al. examined irisin levels in serum and saliva in patients undergoing acute myocardial infarction. Serum and saliva irisin levels were measured at time of presentation and after 72 h, and an increase was observed. The most interesting aspect of that study was that it demonstrated that not only striated muscle in the salivary glands, but also asinary cells are capable of producing various proteins. The fact that the salivary gland and the appendix are luminal organs strengthens our hypothesis of increased irisin secretion due to tension in luminal organs. However, it will also be useful for these organs to be subjected to immunohistochemical examination [10].

Narrowing and obstruction in the lumen trigger inflammation in the physiopathology of diseases in this diagnostic group in which elevated irisin levels are observed. We think that this inflammation can itself affect other autocrine and paracrine organs and increase irisin secretion and that irisin may also be secreted from the lumen. Irisin was once thought to be released only from skeletal muscle but was subsequently shown to also be secreted from other organs [8, 11–13]. We think that the release of irisin can be confirmed by means of immunohistochemical studies in tubular organ diseases.

Although no difference was determined between blood irisin levels in the AA and PA patients in our study, these subjects' irisin levels were higher than those of the control group. Even though irisin cannot detect perforation, it may be considered a good marker of inflammation. We think that the fact that irisin levels rise, irrespective of perforation, in the event of any inflammation in the appendix, a luminal organ, can support a diagnosis of AA.

Infection parameters such as CRP and WBC were higher in AA and PA in our study compared to the nonspecific abdominal pain groups. This finding is in agreement with several studies in the literature, and we think that it can be a valuable one when used together with irisin [14–18].

In conclusion, the differential diagnosis of acute abdominal pains in children and the decision of whether or not to operate can be very difficult and complex. WBC and CRP measurements alone may not be sufficient and reliable. Other diagnostic techniques are therefore needed. The results of this study show that irisin may be a useful diagnostic marker in differentiating AA and PA from other acute abdominal pains.

### 4.1. Limitation

The principal limitation of this study is the low number of patients. Another limitation is that the patients' BMI values were not recorded. There were also various problems in assessing the analytical performance of irisin measured in circulation. The first of these is whether or not the antibodies used in the ELISA method will produce sufficient performance. Albrecht et al. evaluated blood irisin levels in both mouse and human plasma and serum and determined a cross-reaction between some proteins of the same size and ELISA antibodies [19]. They also maintained that irisin, first discovered in 2012, was actually Apolipoprotein A1 (ApoA1), with the same molecular size, measured in plasma. This is still controversial, although there are studies in the literature supporting our findings [4, 7, 20]. Second, Fndc5, a member of the fibronectin type 3 family, is known to be a hormone released after exercise [21]. Although the fact that the exercise status of the patients in this study was not considered is another limitation, that the patients were not performing exercise due to abdominal pain led us to overlook this limitation. There are also various controversies concerning the kits used to determine irisin in serum [22]. The fact that the majority of studies to date have employed first-generation ELISA kits, while a second-generation kit was used in our study, strengthens the reliability of these findings. Stricter confirmatory tests will be useful in terms of preventing the inconsistencies and differing results reported in literature and in achieving a standard in future studies.

## 5. Conclusion

Irisin may be a useful assistant biomarker in the differential diagnosis of acute abdominal pains in children. It may be added to laboratory tests since its use together with WBC and CRP will increase diagnostic accuracy in the diagnosis of AA and PA.

## Figures and Tables

**Table 1:** Distribution of patient.

		**Patient**		**Control**		
		Mean±SD	Min-Max	Mean±SD	Min-Max	p

**Age**		10,0±3,6	2-17	8,0±4,4	1-17	0,003

		n	%	n	%	

**Sex**	Male	70	62,5	29	58	0,587
	Female	42	37,5	21	42	
**Diagnosis**	Acute appendicitis	54	48,2			
	Perforated appendicitis	16	14,3			
	Nonspecific abdominal pain	34	30,4			
	Other abdominal pain	8	7,1			
	AGE	3	1,9			
	MLA	2	1,2			
	FMF	1	0,6			
	UTI	1	0,6			
	Ovarian cyst	1	0,6			

**Table 2:** The groups' mean irisin levels.

	**Irisin**		
	Mean±SD	95% CI Min-Max	Median
**Control**	10.4±6.8	8.4-12.3	8.05
**Acute appendicitis**	13.8±5.7	12.2-15.3	13.7
**Perforated appendicitis**	14.6±5.1	11.8-17.3	15.0
**Non-specific abdominal pain**	8.5±7.9	5.8-11.3	4.5
**Other abdominal pain**	7.8±10.9	-1.3-16.9	3.9
			

**Table 3:** Subgroup analyses for irisin.

	P
Control group vs. Acute Appendicitis	0.001
Control Group vs. Perforated Appendicitis	0.003
Control Group vs. Non-specific abdominal pain	0.013
Control Group vs. Other abdominal pain	0.045
Acute Appendicitis vs. Perforated Appendicitis	0.576
Acute Appendicitis vs. Non-specific abdominal pain	<0.001
Acute Appendicitis vs. Other abdominal pain	0.023
Perforated Appendicitis vs. Non-specific abdominal pain	0.002
Perforated Appendicitis vs. Other abdominal pain	0.043
Non-specific vs. Other abdominal pain	0.478

Bonferroni correction p<0.005.

**Table 4:** WBC and CRP levels.

	**WBC(10** ^**3**^ **/mcL)**		**CRP(0-5mg/L)**	
**Diagnosis**	Mean±SD	Median	Mean±SD	Median
**Acute Appendicitis**	15040.0±3688.7	15450	23.4±58.9	3.7
**Perforated Appendicitis**	18241.3±4865.1	17800	76.2±94.7	26.4
**Non-specific abdominal pain**	10211.2±3821.9	9050	8.0±19.2	0.8
**Other abdominal pain**	14186.3±6723.9	13150	27.6±63.7	4

**Table 5:** Subgroup analyses for WBC and CRP.

	**WBC(10** ^**3**^ **/mcL)**	**CRP(mg/L)**
	P	p
Acute Appendicitis vs. Perforated Appendicitis	0.019	0.029
Acute Appendicitis vs. Non-specific abdominal pain	<0.001	0.001
Acute Appendicitis vs. Other abdominal pain	0.515	0.622
Perforated Appendicitis vs. Non-specific abdominal pain	<0.001	<0.001
Perforated Appendicitis vs. Other abdominal pain	0.126	0.327
Non-specific vs. Other abdominal pain	0.128	0.015

## Data Availability

The data used to support the findings of this study are available from the corresponding author upon request.
